# Prevalence of Low Ankle Brachial Index and Its Association With Pulse Pressure in an Elderly Chinese Population: A Cross-Sectional Study

**DOI:** 10.2188/jea.JE20110140

**Published:** 2012-09-05

**Authors:** Yiqiang Zhan, Jinming Yu, Ruoqing Chen, Yihong Sun, Yuanyuan Fu, Lijun Zhang, Shechang Li, Fen Zhang, Dayi Hu

**Affiliations:** 1 Institute of Clinical Epidemiology, Key Laboratory of Public Health Safety (Ministry of Education), School of Public Health, Fudan University, Shanghai, P. R. China; 2 Heart Center, Peking University People’s Hospital, Beijing, P. R. China

**Keywords:** pulse pressure, ankle brachial index, cross-sectional study

## Abstract

**Background:**

We investigated the prevalence of low ankle brachial index (ABI) and the association of low ABI with pulse pressure among elderly community residents in China.

**Methods:**

This population-based cross-sectional study was conducted in Beijing and recruited 2982 participants who were aged 60 years or older in 2007. Low ABI was defined as an ABI value less than 0.9 in either leg. Participants with or without stroke or coronary heart disease (CHD) were analyzed separately. The association between pulse pressure and low ABI was examined by using multiple logistic regression models.

**Results:**

The prevalence of low ABI was 5.65% (4.24% among men and 6.52% among women; *P* = 0.0221) among participants without stroke or CHD and 10.91% (13.07% among men and 9.49% among women; *P* = 0.1328) among those with stroke or CHD. After adjusting for confounders, the odds ratio (95% CI) for each 5-mm Hg increase in pulse pressure was 1.19 (1.07, 1.33) and 1.10 (1.02, 1.20) for men and women, respectively, among participants without stroke or CHD and 1.17 (1.03, 1.34) and 1.15 (1.02, 1.30) for men and women with stroke or CHD. When pulse pressure was classified into quartiles and the lowest quartile was used as reference, the association between pulse pressure and low ABI remained positive in men and women.

**Conclusions:**

Low ABI was prevalent among elderly Chinese, and pulse pressure was positively associated with low ABI.

## INTRODUCTION

Ankle brachial index (ABI) is used as an indicator in assessing peripheral arterial disease (PAD), which is associated with cardiovascular disease (CVD) events and all-cause mortality,^1^^–^^5^ functional deficiencies, and poor quality of life.^6^^–^^10^ Thus, identification of people with low ABI has important public health implications.

The number of elderly Chinese is increasing rapidly, and the prevalence of low ABI is believed to increase with age. Studies of the prevalence of low ABI in China have usually been confined to specific populations, such as urban residents^11^ and people with hypertension^12^ or diabetes.^13^ Few studies have examined low ABI among a general population of elderly Chinese. In addition, although most traditional and novel CVD risk factors are strongly associated with low ABI,^14^ the association between pulse pressure and low ABI is unclear, especially among elderly Chinese.

In this population-based survey of elderly Chinese, we investigated the prevalence of low ABI and examined the association between pulse pressure and low ABI.

## METHODS

### Study design and participants

This cross-sectional survey of chronic diseases and risk factors was conducted in Beijing from May 2007 through August 2007. Citizens or permanent residents who were 60 years or older were recruited using multistage stratified random sampling. With regard to economic development level, 2 urban administrative districts, 1 urban–rural mixed district, and 1 rural district were selected, after which 38 communities (17 urban communities and 21 rural communities) were randomly sampled. Before the survey, we informed local administrators of the aims and methods of the study. With their help, we were able to disseminate information on our study design via media broadcasts and booklets. On the night before the survey, residents were asked not to drink or eat from 8 PM that night to 8 AM the next day. Ultimately, 3589 community-dwelling adults aged 60 years or older were invited to participate in our survey, and 2998 were recruited (participation rate, 83.53%). Among the recruited participants, we excluded 16 who had disabilities that made them unable to attend physical examinations or interviews; thus, data from 2982 participants were used in the final analysis. All participants provided written informed consent, and ethical approval was obtained from the Ethics Committee of the Beijing Municipal Science and Technology Commission.

### Data collection

A health interview was conducted at community clinics by trained medical staff who used a well-established questionnaire to collect demographic and behavioral information on the study participants. Demographic information included birthday, sex, race, marital status, and education. Behavioral information included smoking status, drinking status, and physical activities. Marital status was classified as married, unmarried, or other (divorced or widowed). Education level was categorized as elementary school or lower (<7 years), middle or high school (7–12 years), and college or higher (>12 years). Physical activity was categorized as 1 hour or longer/day, 2 to 3 hours/day, and 4 hours or longer/day.

The physical examination included anthropometric measurements, blood pressure, medical history, and medication history. Height and weight were measured to the nearest 0.1 cm and 0.1 kg, respectively, with the participant standing barefoot in lightweight clothing. Waist circumference was measured to the nearest 0.1 cm at the midpoint between the 12th rib and the right anterior superior iliac spine. Body mass index (BMI) was calculated as weight (kg) divided by the square of the height (m). Blood pressure was measured using a standard mercury sphygmomanometer on the right arm after the seated participants had rested for 5 minutes. The phase 1 and phase 5 Korotkoff sounds were used to determine systolic blood pressure (SBP) and diastolic blood pressure (DBP), respectively. Blood pressure was measured twice, and the average of the 2 measurements was used in the analysis. Medical history and medication history were obtained from medical records and confirmed by community physicians. All measurements were made by community-licensed physicians.

Blood samples were collected from all participants after an overnight fast. All biochemical measurements were done in the central laboratory of Peking University People’s Hospital. Concentrations of fasting glucose, total cholesterol (TC), high-density lipoprotein cholesterol (HDL-C), triglycerides (TG), and low-density lipoprotein cholesterol (LDL-C) were measured using an autoanalyzer (Hitachi 717, Hitachi Instruments Inc., Tokyo, Japan).

Hypertension was defined as SBP of 140 mm Hg or higher, DBP of 90 mm Hg or higher, or current medication for hypertension. Mean arterial pressure (MAP) was calculated as DBP + ((SBP − DBP)/3). Impaired fasting glucose was defined as a fasting glucose level of 6.1 mmol/L to less than 7.0 mmol/L, and diabetes mellitus was defined as a fasting glucose level of 7.0 mmol/L higher or current medication for diabetes. Dyslipidemia was defined as any of the following: TC greater than 5.18 mmol/L, LDL-C greater than 3.37 mmol/L, HDL-C less than 1.04 mmol/L, or TG greater than 1.70 mmol/L. Central obesity was defined as a waist circumstance greater than 90 cm in men or greater than 85 cm in women.

### Determination of pulse pressure, ABI, and low ABI

Pulse pressure was calculated as the difference between SBP and DBP. ABI was determined by using a standard protocol. After 5 minutes of rest, a standard mercury sphygmomanometer and a Doppler stethoscope (Nicolet, Elite 100R, 5-MHz probe, USA) were used to identify the bilateral brachial, tibial, and dorsal arteries with participants in supine position. Measurements were carried out twice and averaged for analysis. ABI was calculated as the ratio of SBP in the leg to SBP in the ipsilateral arm. The lower ABI value was used as the patient-specific ABI for analysis. Low ABI was defined as an ABI less than 0.9.

### Statistical analysis

Continuous variables are presented as mean ± SD, and categorical variables are presented as frequencies and proportions. In the descriptive analysis, we present the basic characteristics of the study participants and the prevalence of PAD with regard to cardiovascular diseases risk factors. In the exploratory analysis, we used multiple logistic regression models to examine the association between pulse pressure and low ABI in men and women separately. We also examined the multiplicative interaction terms, and the results showed no interaction effect (*P* for interaction ≥0.1 in all tests). Then, to maximize statistical power we analyzed men and women together. Before analyzing pulse pressure as a continuous variable, it was categorized into quartiles, with the lowest quartile as reference. Three models were used in the analysis. The first model included only pulse pressure, the second model adjusted for age (plus sex, when men and women were analyzed together, and the third model adjusted for age, BMI, smoking, LDL-C, HDL-C, physical activity, MAP, community type, and abnormal glucose as confounders (plus sex, when men and women were analyzed together). Odds ratios with 95% CIs are also presented. The diagnostic accuracy of pulse pressure, MAP, SBP, and DBP (on continuous scales) in predicting low ABI was estimated by calculating the area under the curve from receiver operating characteristic curves (AUC–ROC). AUC–ROC values were compared using the algorithm proposed by Delong et al.^15^ All analyses were 2-tailed, and *P* values less than 0.05 was considered statistically significant. All statistics were obtained using SAS 9.2 (SAS Institute, Cary, North Carolina, USA).

## RESULTS

### Basic characteristics of participants

Among the 2982 study participants, the numbers of those with stroke, coronary heart disease (CHD), or both were 263, 367, and 85, respectively. The clinical profiles of those with or without stroke or CHD were different (eTable 1), so we analyzed separately those with or without stroke or CHD. The basic characteristics of study participants without stroke or CHD are shown in Table 1, and 128 (5.65%) had a low ABI: 37 (4.24%) men and 91 (6.52%) women (*P* = 0.0221). Among those with stroke or CHD, 78 (10.91%) had a low ABI: 37 (13.07%) men and 41 (9.49%) women (*P* = 0.1328). The prevalence of low ABI for the total population was 6.91%, 6.41% among men and 7.22% among women (*P* = 0.3908).

**Table 1.** Basic characteristic of study participants without stroke or coronary heart disease

Characteristic	Men (*n* = 872)	Women (*n* = 1395)	*P* value	All (*n* = 2267)
Age (years)	68.0 ± 6.2	67.8 ± 6.0	0.4397	67.9 ± 6.0
Height (cm)	165.55 ± 6.43	153.62 ± 5.75	<0.0001	158.21 ± 8.36
Weight (cm)	66.85 ± 11.22	60.65 ± 10.62	<0.0001	63.03 ± 11.27
BMI (kg/m^2^)	24.34 ± 3.56	25.66 ± 4.07	<0.0001	25.15 ± 3.93
Waist circumference (cm)	88.77 ± 10.23	88.78 ± 10.48	0.9889	88.78 ± 10.38
Hip circumference (cm)	96.37 ± 6.62	98.03 ± 7.99	<0.0001	97.39 ± 7.53
SBP (mm Hg)	135.9 ± 19.8	135.5 ± 19.7	0.6447	135.7 ± 19.8
DBP (mm Hg)	80.0 ± 10.5	78.8 ± 10.1	0.0077	79.3 ± 10.2
Glucose (mmol/l)	5.22 ± 1.59	5.43 ± 1.91	0.0074	5.35 ± 1.80
TC (mmol/l)	4.77 ± 0.85	5.28 ± 0.94	<0.0001	5.09 ± 0.94
TG (mmol/l)	1.33 ± 1.09	1.60 ± 1.07	<0.0001	1.50 ± 1.09
LDL-C (mmol/l)	2.51 ± 0.64	2.78 ± 0.68	<0.0001	2.67 ± 0.68
HDL-C (mmol/l)	1.31 ± 0.32	1.36 ± 0.30	0.0001	1.34 ± 0.31
Pulse pressure (mm Hg)	55.9 ± 17.3	56.7 ± 17.1	0.2920	56.4 ± 17.2
ABI	1.09 ± 0.12	1.05 ± 0.10	<0.0001	1.07 ± 0.11
Marital status			<0.0001	
Unmarried	5 (0.57)	2 (0.14)		7 (0.31)
Other	87 (9.98)	353 (25.30)		440 (19.41)
Married	780 (89.45)	1040 (74.55)		1820 (80.28)
Current smoking			<0.0001	
Yes	581 (66.63)	235 (16.85)		816 (35.99)
No	291 (33.37)	1160 (83.15)		1451 (64.01)
Current drinking			<0.0001	
Yes	377 (43.23)	65 (4.66)		442 (19.50)
No	495 (56.77)	1330 (95.34)		1825 (80.50)
Physical Activity			0.5753	
≤1 hour/day	404 (46.33)	623 (44.66)		1027 (45.30)
2–3 hours/day	416 (47.71)	696 (49.89)		1112 (49.05)
≥4 hours/day	52 (5.96)	76 (5.45)		128 (5.65)
Hypertension	466 (53.44)	793 (56.85)	0.1124	1259 (55.54)
Diabetes	131 (15.02)	240 (17.20)	0.1720	371 (16.37)
Dyslipidemia	441 (50.57)	934 (66.95)	<0.0001	1375 (60.65)
Low ABI	37 (4.24)	91 (6.52)	0.0221	128 (5.65)

Abbreviations: BMI, body mass index; SBP, systolic blood pressure; DBP, diastolic blood pressure; TC, total cholesterol; TG, triglycerides; LDL-C, low-density lipoprotein cholesterol; HDL-C, high-density lipoprotein cholesterol; ABI, ankle brachial index.

### Prevalence of low ABI by age and CVD risk factors

**Figure 1.**
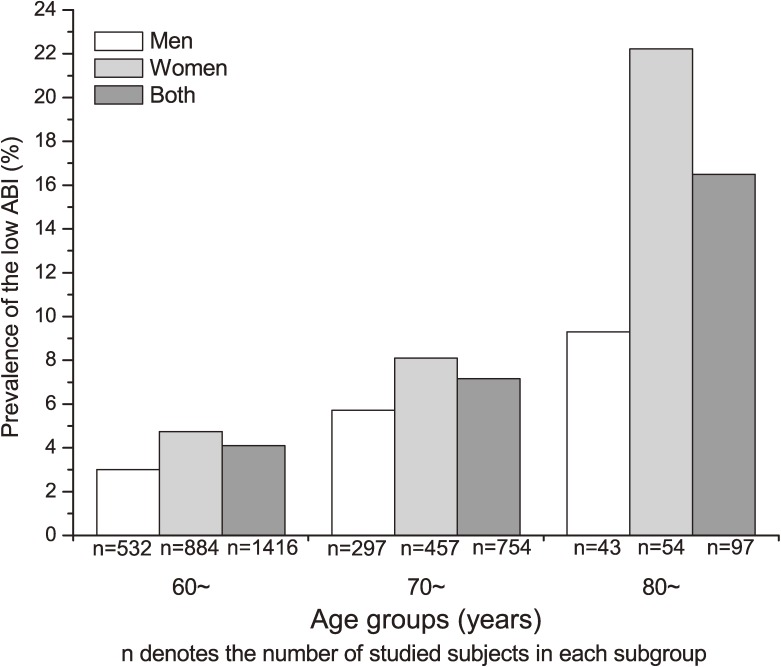
Prevalence of low ankle brachial index (ABI) by age and sex among participants without stroke or coronary heart disease

**Figure 2.**
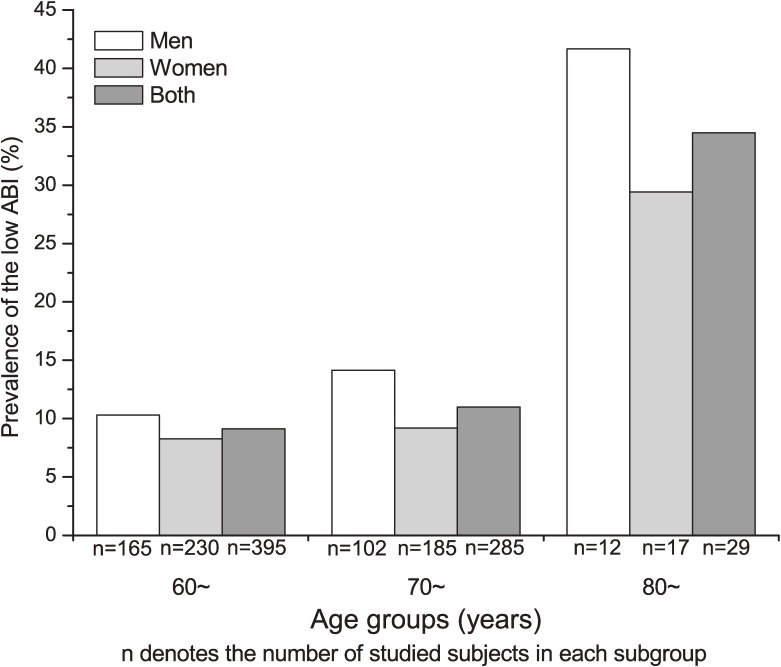
Prevalence of low ankle brachial index (ABI) by age and sex among participants with stroke or coronary heart disease

Figures 1 and 2 illustrate the prevalence of low ABI by age and sex among participants without and with stroke or CHD, respectively. The prevalence of low ABI increased with advancing age in men and women. The distribution of risk factors for low ABI by age group is shown in eTable 2. Women with a current smoking habit, high glucose, high TG, and low HDL-C had a higher prevalence of low ABI than did those without these risk factors. However, among men, the prevalence of low ABI did not significantly differ with regard to risk factors as shown in Table 2.

**Table 2.** Prevalence of low ABI by cardiovascular diseases risk factors among participants without stroke or coronary heart disease

Variable	Men (*n* = 872)	Women (*n* = 1395)	All (*n* = 2267)
Current smoking			
Yes	25 (4.30)	25 (10.64)	50 (6.13)
No	12 (4.12)	66 (5.69)	78 (5.38)
*P* value	0.9015	0.0051	0.4566
Current drinking			
No	31 (6.26)	85 (6.39)	116 (6.36)
Yes	6 (1.59)	6 (9.23)	12 (2.71)
*P* value	0.0007	0.3103	0.0029
Physical activity			
≤1 hour/day	14 (3.47)	37 (5.94)	51 (4.87)
2–3 hours/day	22 (5.29)	50 (7.18)	72 (6.47)
≥4 hours/day	1 (1.92)	4 (5.26)	5 (3.91)
*P* value	0.2998	0.5931	0.2173
Central obesity			
Yes	2 (2.63)	45 (6.47)	47 (6.10)
No	35 (4.40)	45 (6.44)	80 (5.35)
*P* value	0.4657	0.9775	0.4652
Hypertension			
Yes	22 (4.72)	59 (7.44)	81 (6.43)
No	15 (3.69)	32 (5.32)	47 (4.57)
*P* value	0.4532	0.1115	0.0695
Glucose status			
Normal	26 (3.94)	54 (5.33)	80 (4.78)
Impaired fasting glucose	2 (2.47)	10 (7.04)	12 (5.38)
Diabetes mellitus	9 (6.87)	27 (11.25)	36 (9.46)
*P* value	0.2229	0.0037	0.0010
Dyslipidemia			
Yes	19 (4.31)	67 (7.17)	86 (6.19)
No	18 (4.18)	24 (5.21)	42 (4.71)
*P* value	0.9229	0.1616	0.1192
Hypertriglyceridemia			
Yes	6 (3.39)	41 (8.69)	47 (7.10)
No	31 (4.46)	50 (5.42)	81 (5.01)
*P* value	0.5281	0.0193	0.0371
High LDL-C			
Yes	4 (4.65)	20 (8.30)	24 (7.34)
No	33 (4.20)	71 (6.15)	104 (5.36)
*P* value	0.7783	0.2197	0.1516
Low HDL-C			
Yes	10 (6.10)	19 (12.26)	29 (9.09)
No	27 (3.81)	72 (5.81)	99 (5.08)
*P* value	0.1910	0.0022	0.0040
High total cholesterol			
Yes	13 (4.78)	54 (7.45)	67 (6.72)
No	24 (4.00)	37 (5.52)	61 (4.80)
*P* value	0.5968	0.1456	0.0497
Community type			
Rural	14 (3.58)	23 (4.96)	37 (4.33)
Mixed rural–urban	11 (4.35)	33 (7.25)	44 (6.21)
Urban	12 (5.26)	35 (7.35)	47 (6.68)
*P* value	0.6026	0.2464	0.0992

Abbreviations: LDL-C, low-density lipoprotein cholesterol; HDL-C, high-density lipoprotein cholesterol.

### Performance of blood pressure indices as predictors of low ABI

The AUC–ROC values (95% CI) to identify low ABI for pulse pressure, MAP, SBP, and DBP (on continuous scales) were 0.61 (0.56, 0.67), 0.51 (0.45, 0.56), 0.57 (0.52, 0.62), and 0.57 (0.51, 0.63), respectively. The AUC–ROC values for MAP and SBP were smaller than that for pulse pressure (*P* = 0.0181 and *P* = 0.0046, respectively), while the AUC–ROC for DBP did not significantly differ (*P* = 0.2467) from that for pulse pressure.

### Association between pulse pressure and low ABI

Table 3 shows the association between pulse pressure and low ABI among participants without stroke or CHD. Model 1 included only pulse pressure, model 2 was adjusted for age (plus sex, when men and women were analyzed together), and model 3 was adjusted for age, BMI, LDL-C, HDL-C, MAP, community type, physical activity, and high glucose (plus sex, when men and women were analyzed together). The 25th, 50th, and 75th percentiles of pulse pressure were 44.0, 54.0, and 66.0 mm Hg, respectively. In model 3, each 5-mm Hg increase in pulse pressure was associated with a 19%, 10%, and 13% increase in the risk of low ABI among men, women, and all participants, respectively. When pulse pressure was categorized into quartiles and the lowest quartile was used as reference, the risk of low ABI was even higher.

**Table 3.** Association between pulse pressure and low ABI among participants without stroke or coronary heart disease

Pulse pressure measure	Model 1^a^ (OR and 95% CI)			Model 2^b^ (OR and 95% CI)			Model 3^c^ (OR and 95% CI)		
		
	Men (*n* = 872)	Women (*n* = 1395)	Both (*n* = 2267)	Men (*n* = 872)	Women (*n* = 1395)	Both (*n* = 2267)	Men (*n* = 872)	Women (*n* = 1395)	Both (*n* = 2267)
Quartile									
2	1.84 (0.59, 5.73)	1.02 (0.50, 2.10)	1.24 (0.68, 2.27)	1.67 (0.53, 5.21)	1.01 (0.42, 1.81)	1.06 (0.58, 1.95)	1.84 (0.58, 5.89)	1.01 (0.44, 1.98)	1.15 (0.61, 2.15)
3	2.26 (0.76, 6.72)	1.42 (0.72, 2.81)	1.64 (0.93, 2.92)	1.95 (0.65, 5.86)	1.15 (0.57, 2.29)	1.35 (0.75, 2.41)	2.64 (0.84, 8.33)	1.28 (0.62, 2.65)	1.60 (0.87, 2.95)
4	3.43 (1.21, 9.69)	2.64 (1.43, 4.89)	2.90 (1.71, 4.92)	2.41 (0.83, 7.03)	1.84 (0.97, 3.48)	1.99 (1.15, 3.45)	4.24 (1.22, 14.75)	2.28 (1.09, 4.75)	2.73 (1.46, 5.10)
Continuous (per 5-mm Hg increase)	1.14 (1.05, 1.23)	1.14 (1.07, 1.22)	1.12 (1.07, 1.17)	1.10 (1.01, 1.20)	1.07 (1.01, 1.14)	1.08 (1.03, 1.14)	1.19 (1.07, 1.33)	1.10 (1.02, 1.20)	1.13 (1.06, 1.20)

^a^Model 1 included only pulse pressure.

^b^Model 2 included pulse pressure and age (plus sex, when men and women were analyzed together).

^c^Model 3 included pulse pressure, age, body mass index, smoking, low-density lipoprotein cholesterol, high-density lipoprotein cholesterol, physical activity, mean arterial pressure, community type, and abnormal glucose (plus sex, when men and women were analyzed together).

Odds ratios (ORs) were calculated using the first quartile as reference.

Table 4 shows the association between pulse pressure and low ABI among participants with stroke or CHD. The 25th, 50th, and 75th percentiles of pulse pressure were 48.0, 59.5, and 70.0 mm Hg, respectively. In Model 3, each 5-mm Hg increase in pulse pressure was associated with a 17%, 15%, and 15% increase in the risk of low ABI among men, women, and all participants, respectively.

**Table 4.** Association between pulse pressure and low ABI among participants with stroke or coronary heart disease

Pulse pressure measure	Model 1^a^ (OR and 95% CI)			Model 2^b^ (OR and 95% CI)			Model 3^c^ (OR and 95% CI)		
		
	Men (*n* = 283)	Women (*n* = 432)	Both (*n* = 715)	Men (*n* = 283)	Women (*n* = 432)	Both (*n* = 715)	Men (*n* = 283)	Women (*n* = 432)	Both (*n* = 715)
Quartile									
2	1.27 (0.62, 2.62)	1.69 (0.72, 3.98)	1.42 (0.82, 2.45)	1.30 (0.63, 2.68)	1.68 (0.70, 4.04)	1.44 (0.82, 2.51)	1.40 (0.64, 3.05)	2.13 (0.81, 5.58)	1.57 (0.89, 2.80)
3	1.57 (0.77, 3.21)	2.25 (0.98, 5.18)	1.82 (1.06, 3.10)	1.38 (0.67, 2.85)	1.85 (0.78, 4.40)	1.52 (0.88, 2.64)	1.42 (0.65, 3.12)	2.43 (0.93, 6.32)	1.64 (0.92, 2.92)
4	1.79 (0.85, 3.78)	4.06 (1.83, 9.02)	2.74 (1.61, 4.64)	1.73 (0.82, 3.68)	2.91 (1.28, 6.62)	2.33 (1.36, 3.99)	2.58 (1.04, 6.39)	5.72 (2.13, 15.38)	2.57 (1.40, 4.71)
Continuous (per 5-mm Hg increase)	1.10 (0.99, 1.22)	1.14 (1.03, 1.25)	1.11 (1.04, 1.18)	1.09 (0.99, 1.21)	1.07 (0.98, 1.17)	1.08 (1.01, 1.16)	1.17 (1.03, 1.34)	1.15 (1.02, 1.30)	1.15 (1.05, 1.25)

^a^Model 1 included only pulse pressure.

^b^Model 2 included pulse pressure and age (plus sex, when men and women were analyzed together).

^c^Model 3 included pulse pressure, age, body mass index, smoking, low-density lipoprotein cholesterol, high-density lipoprotein cholesterol, physical activity, mean arterial pressure, community type, and abnormal glucose (plus sex, when men and women were analyzed together).

Odds ratios (ORs) were calculated using the first quartile as the reference group.

## DISCUSSION

Although several studies have examined the prevalence of low ABI in China, the source populations differed. Most studies focused on a specific population, such as urban residents,^11^ people attending cardiovascular clinics,^12^ or people with diabetes.^13^ The present study investigated population-based communities in both urban and rural regions of China and focused on individuals aged 60 years or older, who are at higher risk of developing cardiovascular diseases. The study participants were randomly selected from residential communities, and the prevalence of low ABI, and the association of low ABI with pulse pressure, can be generalized to the general elderly population of Beijing, China.

The prevalence of low ABI in the present study was lower than that in another study carried out in Beijing,^11^ which reported a prevalence of 15.3%. The discrepancy may be due to differences in source population, study period, and diagnostic criteria. However, the prevalence of low ABI among people with hypertension in our study was similar to the prevalence of 8.7% reported among hypertensive individuals in a community in central China.^12^ Additionally, the prevalence of low ABI in our study was much lower than in Western countries. The Rotterdam Study reported a PAD prevalence of 19.1%,^16^ and a French study reported that the prevalence of PAD ranged from 10.4% to 38.0% in people with cardiovascular risk factors.^17^ Although PAD was only found in 7.6% of the general population in Spain, it was diagnosed in 17% of those with diabetes.^18^ The result was similar in an Italian study, which reported that PAD was present in 17% of people with diabetes.^14^ In contrast, the Tanno-Sobetsu Study in Japan found that the prevalence of arteriosclerosis obliterans was only 3.4% among rural residents aged 65 years or older.^19^ The Kyushu and Okinawa Population Study (KOPS) reported that the prevalence of PAD was 2.3% among a general population of adults aged 60 years or older.^20^ Differences in recruitments methods, distributions of risk factors, socioeconomic status, and lifestyle might explain these variations.

In this study, among participants without stroke or CHD, the prevalence of low ABI was higher among women than among men, which was consistent with the findings of other studies^11^^,^^13^^,^^14^^,^^18^; however, some studies observed no such sex difference.^12^^,^^21^^,^^22^ Likewise, we observed no sex difference in the prevalence of low ABI among participants with stroke or CHD. Nevertheless, among those without stroke or CHD, the prevalence of low ABI was higher among women with CVD risk factors than among those without these risk factors, and the prevalence was higher for urban than rural regions. In addition, older participants had a higher risk for low ABI. In our analysis of which continuous-scaled blood pressure indices were more strongly associated with low ABI, the AUC–ROC showed that the diagnostic ability of pulse pressure was better than those of MAP and SBP in predicting low ABI.

We hypothesized that pulse pressure is positively associated with low ABI, and our findings do not contradict this hypothesis. Our results were consistent between participants with and without stroke or CHD. After adjustment for age and sex, the association between pulse pressure and low ABI remained significant in men and women, and the results were consistent even after adjusting for traditional CVD risk factors. A few studies examined the association of pulse pressure and low ABI in specific populations and found that pulse pressure was an important predictor of low ABI.^14^^,^^23^^–^^25^ Our results are in accordance with the findings of previous studies, but our results were based on a general population rather than populations with a high risk of CVD.

Several possible mechanisms could explain the link between pulse pressure and low ABI. Pulse pressure was shown to damage endothelial function in people with^26^ or without CVD,^27^^,^^28^ and endothelial damage is a sign of atherosclerosis,^29^^,^^30^ which can cause arterial stiffness.^31^^,^^32^ In addition, pulse pressure was found to be related to left ventricular hypertrophy,^33^ which can reduce myocardial oxygen supply^34^ and lead to deficient peripheral oxygen supply. Finally, a bidirectional cause-and-effect relationship might partially account for the positive association between pulse pressure and low ABI, as higher pulse pressure is both a cause and a consequence of atherosclerosis.^35^ Higher pulse pressure can be induced by arterial stiffness due to increasing wave reflection,^36^^,^^37^ which could explain the statistical association between pulse pressure and low ABI and suggests the possibility of a vicious circle.

Because the present data were collected in a cross-sectional study, we cannot make causal inferences regarding whether an individual’s higher pulse pressure preceded low ABI. Furthermore, the potential bias caused by nonresponse to the survey is a concern, as is the fact that more women than men were recruited in our study. Women paid much more attention to their health, which could have resulted in overestimation of the prevalence of low ABI in the general population.

In conclusion, low ABI was prevalent among elderly Chinese, especially those with stroke or CHD. Pulse pressure was associated with low ABI, and this association might be mediated by several mechanisms.

## ONLINE ONLY MATERIALS

eTables. eTables are available on the journal’s website at http://dx.doi.org/10.2188/jea.JE20110140.

## References

[1] Resnick HE, Lindsay RS, McDermott MM, Devereux RB, Jones KL, Fabsitz RR, Relationship of high and low ankle brachial index to all-cause and cardiovascular disease mortality: the Strong Heart Study. Circulation. 2004;109:733–9 10.1161/01.CIR.0000112642.63927.54 14970108

[2] Golomb BA, Dang TT, Criqui MH Peripheral arterial disease: morbidity and mortality implications. Circulation. 2006;114:688–99 10.1161/CIRCULATIONAHA.105.593442 16908785

[3] Sutton-Tyrrell K, Venkitachalam L, Kanaya AM, Boudreau R, Harris T, Thompson T, Relationship of ankle blood pressures to cardiovascular events in older adults. Stroke. 2008;39:863–9 10.1161/STROKEAHA.107.487439 18258843

[4] Luo Y, Li X, Li J, Wang X, Xu Y, Qiao Y, Peripheral arterial disease, chronic kidney disease, and mortality: the Chinese Ankle Brachial Index Cohort Study. Vasc Med. 2010;15:107–12 10.1177/1358863X09357230 20133341

[5] Criqui MH, McClelland RL, McDermott MM, Allison MA, Blumenthal RS, Aboyans V, The ankle-brachial index and incident cardiovascular events in the MESA (Multi-Ethnic Study of Atherosclerosis). J Am Coll Cardiol. 2010;56:1506–12 10.1016/j.jacc.2010.04.060 20951328 PMC2962558

[6] Nehler MR, McDermott MM, Treat-Jacobson D, Chetter I, Regensteiner JG Functional outcomes and quality of life in peripheral arterial disease: current status. Vasc Med. 2003;8:115–26 10.1191/1358863x03vm483ra 14518614

[7] Dumville JC, Lee AJ, Smith FB, Fowkes FG The health-related quality of life of people with peripheral arterial disease in the community: the Edinburgh Artery Study. Br J Gen Pract. 2004;54:826–31 15527608 PMC1324915

[8] Killewich LA Improving functional status and quality of life in elderly patients with peripheral arterial disease. J Am Coll Surg. 2006;202:345–55 10.1016/j.jamcollsurg.2005.09.026 16427563

[9] Liles DR, Kallen MA, Petersen LA, Bush RL Quality of life and peripheral arterial disease. J Surg Res. 2006;136:294–301 10.1016/j.jss.2006.06.008 17046794

[10] Regensteiner JG, Hiatt WR, Coll JR, Criqui MH, Treat-Jacobson D, McDermott MM, The impact of peripheral arterial disease on health-related quality of life in the Peripheral Arterial Disease Awareness, Risk, and Treatment: New Resources for Survival (PARTNERS) Program. Vasc Med. 2008;13:15–24 10.1177/1358863X07084911 18372434

[11] He Y, Jiang Y, Wang J, Fan L, Li X, Hu FB Prevalence of peripheral arterial disease and its association with smoking in a population-based study in Beijing, China. J Vasc Surg. 2006;44:333–8 10.1016/j.jvs.2006.03.032 16890864

[12] Yang X, Sun K, Zhang W, Wu H, Zhang H, Hui R Prevalence of and risk factors for peripheral arterial disease in the patients with hypertension among Han Chinese. J Vasc Surg. 2007;46:296–302 10.1016/j.jvs.2007.03.034 17600667

[13] Li J, Hasimu B, Yu J, Wang J, Hu D Prevalence of peripheral arterial disease and risk factors for the low and high ankle-branchial index in Chinese patients with type 2 diabetes. J Health Sci. 2006;52:97–102 10.1248/jhs.52.97

[14] Bianchi C, Penno G, Pancani F, Civitelli A, Piaggesi A, Caricato F, Non-traditional cardiovascular risk factors contribute to peripheral arterial disease in patients with type 2 diabetes. Diabetes Res Clin Pract. 2007;78:246–53 10.1016/j.diabres.2007.03.020 17498833

[15] DeLong ER, DeLong DM, Clarke-Pearson DL Comparing the areas under two or more correlated receiver operating characteristic curves: a nonparametric approach. Biometrics. 1988;44:837–45 10.2307/2531595 3203132

[16] Meijer WT, Hoes AW, Rutgers D, Bots ML, Hofman A, Grobbee DE Peripheral arterial disease in the elderly: The Rotterdam Study. Arterioscler Thromb Vasc Biol. 1998;18:185–92 10.1161/01.ATV.18.2.185 9484982

[17] Cacoub P, Cambou JP, Kownator S, Belliard JP, Beregi JP, Branchereau A, Prevalence of peripheral arterial disease in high-risk patients using ankle-brachial index in general practice: a cross-sectional study. Int J Clin Pract. 2009;63:63–70 10.1111/j.1742-1241.2008.01953.x 19125994 PMC2705819

[18] Alzamora MT, Forés R, Baena-Díez JM, Pera G, Toran P, Sorribes M, The peripheral arterial disease study (PERART/ARTPER): prevalence and risk factors in the general population. BMC Public Health. 2010;10:38 10.1186/1471-2458-10-38 20529387 PMC2835682

[19] Fujiwara T, Saitoh S, Takagi S, Ohnishi H, Ohata J, Takeuchi H, Prevalence of asymptomatic arteriosclerosis obliterans and its relationship with risk factors in inhabitants of rural communities in Japan: Tanno-Sobetsu study. Atherosclerosis. 2004;177:83–8 10.1016/j.atherosclerosis.2004.05.028 15488869

[20] Ohnishi H, Sawayama Y, Furusyo N, Maeda S, Tokunaga S, Hayashi J Risk factors for and the prevalence of peripheral arterial disease and its relationship to carotid atherosclerosis: the Kyushu and Okinawa Population Study (KOPS). J Atheroscler Thromb. 2010;17:751–8 10.5551/jat.3731 20523009

[21] Collins TC, Suarez-Almazor M, Bush RL, Petersen NJ Gender and peripheral arterial disease. J Am Board Fam Med. 2006;19:132–40 10.3122/jabfm.19.2.132 16513901

[22] Bozkurt AK, Tasci I, Tabak O, Gumus M, Kaplan Y Peripheral artery disease assessed by ankle-brachial index in patients with established cardiovascular disease or at least one risk factor for atherothrombosis—CAREFUL Study: A national, multi-center, cross-sectional observational study. BMC Cardiovasc Disord. 2011;11:4 10.1186/1471-2261-11-4 21247449 PMC3033857

[23] Tseng CH Pulse pressure as a risk factor for peripheral vascular disease in type 2 diabetic patients. Clin Exp Hypertens. 2003;25:475–85 10.1081/CEH-120025331 14649305

[24] Subramaniam T, Nang EE, Lim SC, Wu Y, Khoo CM, Lee J, Distribution of ankle–brachial index and the risk factors of peripheral artery disease in a multi-ethnic Asian population. Vasc Med. 2011;16:87–95 10.1177/1358863X11400781 21447605

[25] Heffernan KS, Ranadive S, Weikert M, Lane A, Yan H, Fernhall B, Pulse pressure is associated with walking impairment in multiple sclerosis. J Neurol Sci. 2011;309:105–9 10.1016/j.jns.2011.07.004 21821264

[26] Raamat R, Jagomägi K, Talts J, Toska K, Walløe L Beat-to-beat measurement of the finger arterial pressure pulse shape index at rest and during exercise. Clin Physiol Funct Imaging. 2003;23:87–91 10.1046/j.1475-097X.2003.00474.x 12641602

[27] Beigel R, Dvir D, Arbel Y, Shechter A, Feinberg MS, Shechter M Pulse pressure is a predictor of vascular endothelial function in middle-aged subjects with no apparent heart disease. Vasc Med. 2010;15:299–305 10.1177/1358863X10373300 20724375

[28] McEniery CM, Wallace S, Mackenzie IS, McDonnell B, Yasmin, Newby DE, Endothelial function is associated with pulse pressure, pulse wave velocity, and augmentation index in healthy humans. Hypertension. 2006;48:602–8 10.1161/01.HYP.0000239206.64270.5f 16940223

[29] Davignon J, Ganz P Role of endothelial dysfunction in atherosclerosis. Circulation. 2004;109(23 Suppl 1):III27–32 10.1161/01.CIR.0000131515.03336.f8 15198963

[30] Sitia S, Tomasoni L, Atzeni F, Ambrosio G, Cordiano C, Catapano A, From endothelial dysfunction to atherosclerosis. Autoimmun Rev. 2010;9:830–4 10.1016/j.autrev.2010.07.016 20678595

[31] van Popele NM, Grobbee DE, Bots ML, Asmar R, Topouchian J, Reneman RS, Association between arterial stiffness and atherosclerosis: The Rotterdam study. Stroke. 2001;32:454–60 10.1161/01.STR.32.2.454 11157182

[32] Wykretowicz A, Gerstenberger P, Guzik P, Milewska A, Krauze T, Adamska K, Arterial stiffness in relation to subclinical atherosclerosis. Eur J Clin Invest. 2009;39:11–6 10.1111/j.1365-2362.2008.02057.x 19087126

[33] Toprak A, Reddy J, Chen W, Srinivasan S, Berenson G Relation of pulse pressure and arterial stiffness to concentric left ventricular hypertrophy in young men (from the Bogalusa Heart Study). Am J Cardiol. 2009;103:978–84 10.1016/j.amjcard.2008.12.011 19327426

[34] Neill WA, Fluri-Lundeen JH Myocardial oxygen supply in left ventricular hypertrophy and coronary heart disease. Am J Cardiol. 1979;44:746–53 10.1016/0002-9149(79)90297-2 158305

[35] Dart AM, Kingwell BA Pulse pressure—a review of mechanisms and clinical relevance. J Am Coll Cardiol. 2001;37:975–84 10.1016/S0735-1097(01)01108-1 11263624

[36] Safar ME, Levy BI, Struijker-Boudier H Current perspectives on arterial stiffness and pulse pressure in hypertension and cardiovascular diseases. Circulation. 2003;107:2864–9 10.1161/01.CIR.0000069826.36125.B4 12796414

[37] Mitchell GF, Parise H, Benjamin EJ, Larson MG, Keyes MJ, Vita JA, Changes in arterial stiffness and wave reflection with advancing age in healthy men and women: the Framingham Heart Study. Hypertension. 2004;43:1239–45 10.1161/01.HYP.0000128420.01881.aa 15123572

