# Supplementary material for: Prevalence of Low Ankle Brachial Index and Its Association With Pulse Pressure in an Elderly Chinese Population: A Cross-Sectional Study
Source: J Epidemiol. 2012 Sep 5;22(5):454–61. doi: 10.2188/jea.JE20110140 (PMC3798641; doi:10.2188/jea.JE20110140)
Supplement: eTables. — eTables are available on the journal’s website at http://dx.doi.org/10.2188/jea.JE20110140. [file je-22-454-s001.pdf]

eTable 1. Clinical Profile of Participants With or Without Stroke or Coronary Heart Disease

Variable	With stroke or coronary heart diseases (n=715)	Without stroke or coronary heart diseases (n=2267)	*P* value
Age (years)	68.9± 5.9	67.9± 6.0	0.0002
Height (cm)	158.20± 8.03	158.21± 8.36	0.9680
Weight (cm)	64.71±11.14	63.03±11.27	0.0005
BMI (kg/m^2)	25.80± 3.71	25.15± 3.93	0.0001
Waist (cm)	90.84±10.15	88.78±10.38	<0.0001
Hip (cm)	98.52± 7.62	97.39± 7.53	0.0005
SBP (mm Hg)	139.1±19.8	135.7±19.8	<0.0001
DBP (mm Hg)	79.5±10.7	79.3±10.2	0.5566
Glucose (mmol/l)	5.53± 1.91	5.35± 1.80	0.0168
TC (mmol/l)	5.11± 1.03	5.09± 0.94	0.5648
TG (mmol/l)	1.66± 1.06	1.50± 1.09	0.0003
LDL-C (mmol/l)	2.66± 0.73	2.67± 0.68	0.7383
HDL-C (mmol/l)	1.30± 0.33	1.34± 0.31	0.0005
Pulse pressure (mm Hg)	59.5±17.2	56.4±17.2	<0.0001
ABI	1.04± 0.13	1.07± 0.11	<0.0001
Hypertension	530 (74.13)	1259 (55.54)	<0.0001
Diabetes	195 (27.27)	371 (16.37)	<0.0001
Dyslipidemia	487 (68.11)	1375 (60.65)	<0.0001
Low ABI	78 (10.91)	128 (5.65)	<0.0001

Abbreviations: BMI: body mass index, SBP: systolic blood pressure, DBP: diastolic blood pressure, TC: total cholesterol, TG: triglycerides, LDL-C: low-density lipoprotein cholesterol, HDL-C: high-density lipoprotein cholesterol, ABI: ankle brachial index.

eTable 2. Distribution of Risk Factors for Low ABI by Sex and Age Group

Variable	Men			*P*	Women			*P*	Both			*P*
	60-69	70-79	80-		60-69	70-79	80-		60-69	70-79	80-	
Current smoking	376(70.68)	180(60.61)	25(58.14)	0.0021	144(16.29)	76(16.67)	14(25.93)	0.2131	520(36.72)	256(34.0)	39(40.21)	0.6345
Current drinking	256(48.12)	110(37.04)	11(25.58)	<0.0001	36(4.07)	26(5.70)	3(5.56)	0.2015	292(20.62)	136(18.06)	14(14.43)	0.0541
Obesity	39(7.33)	33(11.11)	4(9.30)	0.1193	432(48.87)	235(51.54)	28(51.85)	0.3517	471(33.26)	268(35.59)	32(32.99)	0.4493
Hypertension	281(52.82)	158(53.20)	27(62.79)	0.3972	462(52.26)	298(65.35)	33(61.11)	<0.0001	743(52.47)	456(60.56)	60(61.86)	0.0003
Fasting glucose												
Normal	406(76.32)	220(74.07)	34(79.07)	0.7999	647(73.19)	331(72.59)	35(64.81)	0.3233	1053(74.36)	551(73.17)	69(71.13)	0.3771
IFG	52(9.77)	27(9.09)	2(4.65)	0.3633	90(10.18)	43(9.43)	9(16.67)	0.5842	142(10.03)	70(9.30)	11(11.34)	0.8776
DM	74(13.91)	50(16.84)	7(16.28)	0.2970	147(16.63)	82(17.98)	10(18.52)	0.4662	221(15.61)	132(17.53)	17(17.53)	0.2405
Abnormal TC	177(33.27)	87(29.29)	8(18.60)	0.0404	459(51.92)	241(52.85)	24(44.44)	0.7297	636(44.92)	328(43.56)	32(32.99)	0.0776
Abnormal TG	113(21.24)	60(20.20)	4(9.30)	0.1625	323(36.54)	129(28.29)	19(35.19)	0.0202	436(30.79)	189(25.1)	23(23.71)	0.0041
Abnormal LDL-C	57(10.71)	24(8.08)	5(11.63)	0.4656	156(17.65)	79(17.32)	6(11.11)	0.4068	213(15.04)	103(13.68)	11(11.34)	0.2209
Abnormal HDL-C	89(16.73)	68(22.90)	7(16.28)	0.1413	103(11.65)	40(8.77)	12(22.22)	0.8536	192(13.56)	108(14.34)	19(19.59)	0.1844
Dyslipidemia	272(51.13)	153(51.52)	16(37.21)	0.3159	600(67.87)	296(64.91)	37(68.52)	0.4623	872(61.58)	449(59.63)	53(54.64)	0.1546

Abbreviations: IFG: impaired fasting glucose; DM: diabetes mellitus; TC: total cholesterol, TG: triglycerides, LDL-C: low-density lipoprotein cholesterol, HDL-C: high-density lipoprotein cholesterol.

P values were calculated using the Cochran–Armitage Trend Test.
